# Measurement of the Poisson expansion effect on crack openings in self-sensing concrete

**DOI:** 10.1038/s41598-025-04135-9

**Published:** 2025-07-01

**Authors:** Xueying Wang, Lee Scott Cunningham, Michele Win Tai Mak, Janet Lees, Abir Al-Tabbaa, Stuart Kenneth Haigh

**Affiliations:** 1 Department of Engineering, University of Cambridge, Cambridge, CB2 1PZ UK; 2 Department of Civil Engineering and Management, University of Manchester, Manchester, M13 9PL UK; 3 School of Civil Engineering, University of Leeds, Leeds, LS2 9JT UK

**Keywords:** Degradation, Finite element modelling, Infrastructure management, Measurement technique, Non-destructive testing, Structural health monitoring, Engineering, Materials science

## Abstract

Concrete infrastructure tends to degrade with extended service life, but detecting deterioration with conventional inspection methods can be challenging. Existing approaches such as developing self-sensing concrete by adding electrically conductive fillers to the cement matrix often suffer from high costs and complex manufacturing processes for casting the concrete from fresh. This study looks beyond the commonly discussed resistance-based and capacitance-based self-sensing mechanisms in the elastic deformation regime of the concrete and investigates the changes in electrical conductivity due to micro-crack opening through Poisson expansion by utilising the measurement technique of drilling the concrete to insert conductive epoxy for electrode embedment to achieve intrinsic self-sensing on as-built regular concrete (i.e. without conductive fillers). Experimental results on existing plain concrete samples showed a good correlation between the compressive load and the fractional change in resistivity (FCR). Finite element analysis (FEA) was then used to further investigate the effect of changes in conductivity in the direction of loading and orthogonal directions on the measured electrical behaviour of the concrete. Results showed that the correlation between the FCR and the compressive load depends on the relationship between the load-parallel and -orthogonal gauge factors. Beyond a threshold level, the electrical signal picked up by the electrodes reflects the material’s behaviour in the direction orthogonal to the loading direction due to Poisson expansion. Further numerical simulation has suggested that to achieve a stable measurement, the electrodes’ penetration should equal the spacing.

## Introduction

Concrete is the most extensively used construction material around the world, however, many concrete structures, built in the mid to late 20th century, were not designed or constructed for current load demands and exhibit degradation of performance. The design of concrete structures is nowadays based on safety and serviceability criteria prescribed in codes of practice. These criteria involve allowable stresses and deflections predicted using structural analysis. If the stress within a structural member exceeds the limit value, the integrity and safety of the structure can be compromised. If the deflection of a structural member approaches or exceeds the serviceability limit, the intended function of the structure might be impaired. Consequently, assessing deflections and damage in concrete structures represents important objectives of strain-based Structural Health Monitoring (SHM). Monitoring of the structural health of existing buildings typically utilises sensors to monitor structural deflections under working loads. While these give indications of the changes in structural performance over time, they do not explicitly measure the stress and damage state of the concrete. In the last few decades, scientists have been searching for alternatives to create a more resilient and sustainable sensing method. One of the biggest achievements is the material revolution of Intrinsic Self-Sensing Concrete (ISSC), a smart construction material which can sense its own deformation and damage. The sensing mechanism for ISSC is achieved by combining electrically conductive fillers such as carbon nanomaterials and concrete to realise a sensing function by measuring the change of electrical properties within the material itself. The initial conception of self-sensing concrete was introduced by Chen and Chung^1^ in the US in the 1990s, who also proposed the concept of a ‘smart concrete structure’ and resistance-based self-sensing concrete. After further studies and experimental demonstration, they discovered that the composite of concrete and carbon fibre can act as a strain and stress sensor. Since then, ISSC has gained great attention from researchers all over the world, and research findings are increasing year by year. However, ISSC not only suffers from the relatively high cost of conductive fillers as well as the complex manufacturing process to embed the functional fillers but is also limited to newly-built structures. Finding an approach to effectively monitor the structural health of as-built concrete structures is therefore key to ensure widespread applicability. This notion takes on even more significance given the environmental imperative to extend the life of the existing built environment. Recent studies in the self-sensing concrete field have reported another sensing mechanism, i.e., capacitance-based structural self-sensing^2^, on newly made plain cementitious composites (hydrated paste, mortar or concrete without any conductive fillers) with electrodes embedded into the specimens while casting. Zhang et al.^3^ tested plain mortar specimens after 28 days of water curing with cyclic compressive loading and found the fractional change in resistivity (FCR) decreased as the compressive stress/strain increased, which indicated piezoresistive property. They also reported the stress and strain sensitivity of the specimens firstly increased then decreased with increasing content of carbon nanotube and nano carbon black composite fillers. Ding et al.^4^ measured the fractional change in resistivity of newly made plain cement paste samples under both monotonic and cyclic compressive loading and found piezoresistive property with a gauge factor of 21.5 and the stress sensitivity being 0.11%/MPa. A carbon nanotube addition in the composites was also reported to evidently enlarge the FCR, indicating a higher sensitivity. Meoni et al.^5^ investigated the strain sensing capability of newly made plain cement paste and concrete specimens through compressive tests with quasi-static loads. The results showed both the paste and concrete samples without nanotubes exhibited clear strain sensitivity with a significant non-linear relationship between the relative change in electrical resistivity and the applied deformation. This was more evident in the concrete sample where the response of the base material also exhibited a hysteresis. A similar phenomenon was also observed by Wang et al.^6^, where clear stress/strain sensitivities with hysteresis were reported on newly made plain cement paste samples. While previous studies present evidence on achieving self-sensing by embedding electrodes into fresh plain cementitious composites during casting^7^, this paper for the first time looks beyond the commonly discussed resistance-based and capacitance-based self-sensing mechanisms only in the elastic deformation regime of the concrete and explores the sensing behaviour of as-built plain concrete with micro-cracks already developed in the material. Through experiments, finite element modelling (FEM) and mathematical analysis, the possibility of achieving self-sensing on existing concrete structures without the addition of electrically conductive fillers by utilising the changes in electrical conductivity due to crack opening through Poisson expansion will be discussed. This paper is structured as follows: Sect “Materials and experimental methodology” describes the materials and experimental methods used in this study. Sect “Experiment results of the concrete cylinder samples” presents the experimental results and discussion which was carried out by testing concrete cylinders cored from an existing concrete beam under cyclic compressive loading. Sect “Numerical and mathematical model description and validation” focuses on the numerical and mathematical modelling description and validation, in which Sect “Numerical investigation on the anisotropic conductivity” uses FEM to investigate the influence of anisotropic conductivity due to crack opening; Sect “Mathematical model description and validation” provides mathematical analysis to validate the proposed theory on the sensing mechanism of the tested samples; Sect “Numerical investigation on effects of sample geometry and electrode placement” uses FEM to further predict and optimise the electrical responses of different sample geometries and electrode configurations. Sect “Conclusion” concludes the paper and outlines directions for future research.

## Materials and experimental methodology

To test the possibility of achieving self-sensing on existing concrete structures without adding any electrically conductive fillers, an existing concrete beam was used to simulate real-world application conditions. This concrete beam was cast in December 2022 in a plywood formwork, compacted with a poker vibrator and a pneumatic hammer in three horizontal layers and covered with plastic sheets immediately after casting. Table 1 summarises the composition of the mix and quantities of the constituents. The w/c ratio of this mix was 0.4.

**Table 1:** The composition of the concrete mix and quantities of the constituents.

Constituent	Type	Assumed density (kg/m^3^)	Amount of the constituents (kg/m^3^)
Water	-	1000	220
Cement	CEM I 52.5 N	3100	550
Fine aggregate	0/4 mm	2535	700
Coarse aggregate (uncrushed)	4/10 mm	2535	855

**Fig. 1:**
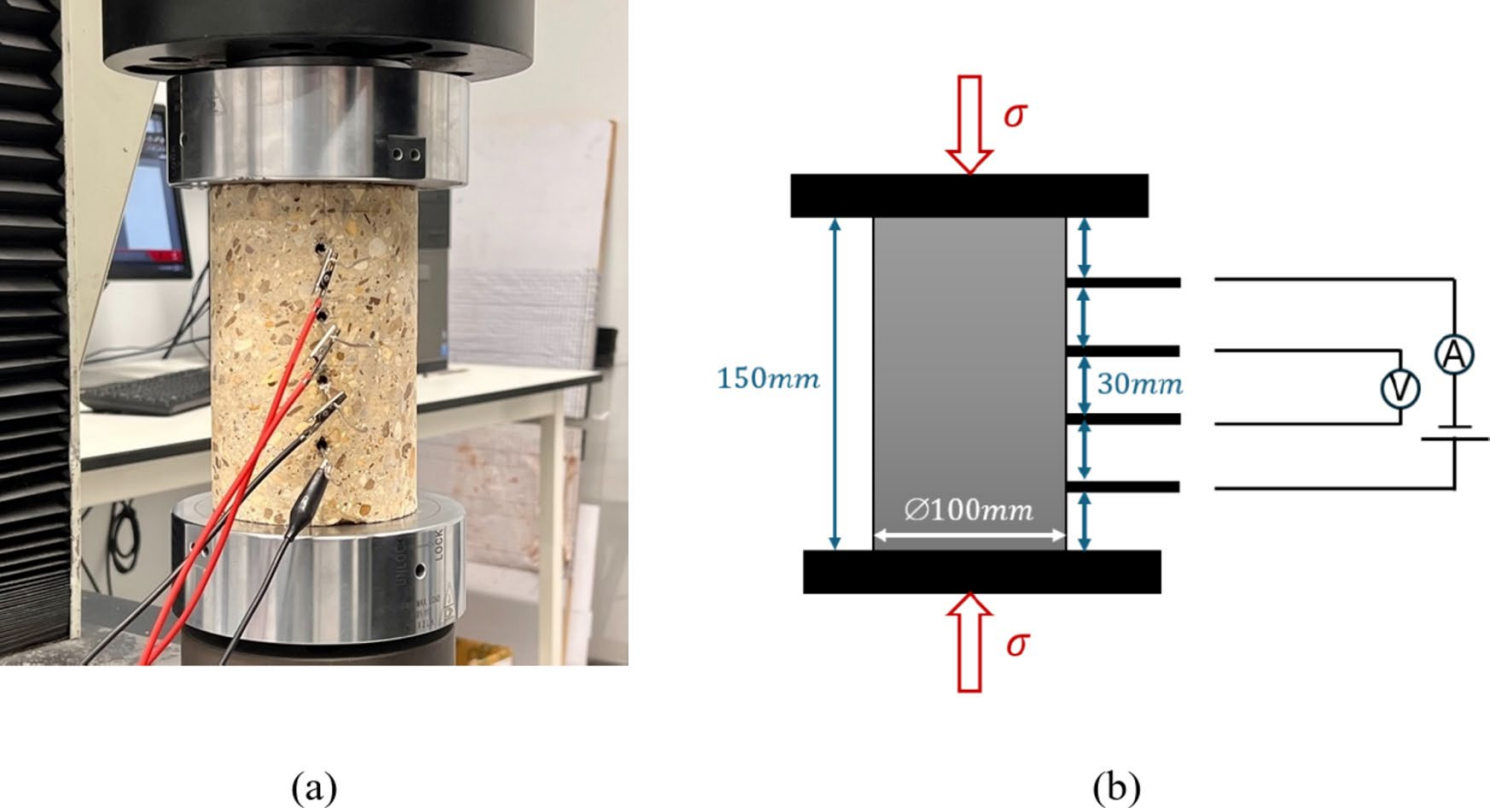
The cylinder cored from the concrete beam for cyclic compressive piezo-resistivity tests (**a**) a photo (**b**) a schematic diagram with annotations.

Approximately 24 h after casting, the specimen was demoulded, covered with plastic sheets and left to cure in an indoor laboratory environment for 28 days. The curing conditions of temperature and humidity were not actively controlled or monitored. The 28 days’ average compressive strength was 61.1 MPa for cylinder testing and 69.0 MPa for cube testing. After 28 days, the structural specimen was left uncovered in an outdoor environment until the cylinders were cored 14 months later. The cored concrete cylinders have a diameter of 100 mm and a height of 150 mm. To create smooth and parallel surfaces for uniformly spread compressive loading, the top and bottom of the cylinders were ground using a concrete grinder. To measure the electrical properties of the specimen in the least destructive way, wire-form electrodes were embedded instead of sheet-form electrodes due to their penetration area with the specimen, even though they tend to have more concentrated current flow, and a less uniform distribution compared to sheet-form electrodes. To embed wire-form electrodes for electrical properties measurement, four equally spaced ∅7 mm holes were drilled along the loading direction on the side of the cylinder to a depth of 50 mm. Electrically conductive epoxy (8331D silver conductive epoxy adhesive from MG Chemicals) was then used to attach copper wire electrodes into the holes in the samples. Figure 1 presents the cylinder used for this test. This electrical configuration enables the measurement of piezo-resistivity on as-built concrete structures with the least damage to their structural integrity. However, it requires superior drilling techniques and strong adhesion from the conductive epoxy, which could be challenging.

## Experimental results, numerical modelling and mathematical investigations

### Experiment results of the concrete cylinder samples

**Fig. 2:**
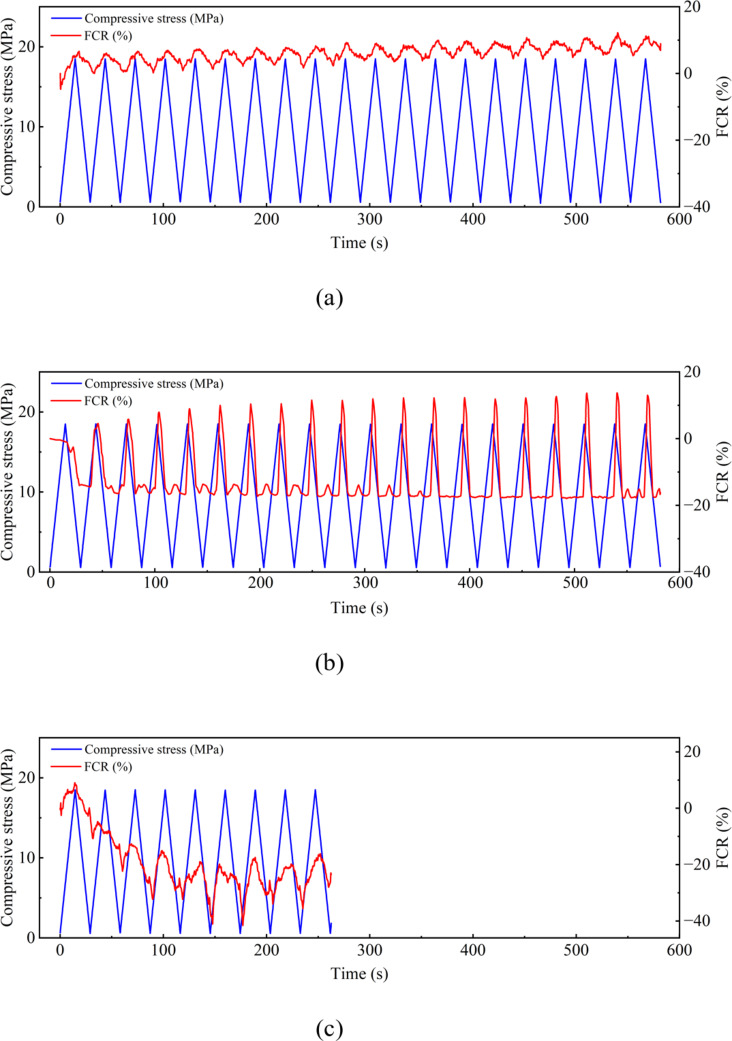
Cyclic compressive test results on three concrete cylinders cored from the concrete beam.

A load-controlled compressive loading machine (INSTRON electro-mechanical universal testing machine 150 kN) was used to apply the loading, and two multimeters and a DC power supply were used to measure the electrical properties of the sample through the 4-probe method^8^. 20 cycles of compressive load with a minimum load of 5 kN and a maximum load of 145 kN were applied to each sample. The maximum compressive stress applied to the sample was around 30% of the compressive strength, equating to a factor of safety of 3 on collapse. Although this would normally fall into the elastic range of concrete, micro-cracks will exist within the concrete sample which will open during loading. This may be exacerbated by additional cracking caused by the drilling of the holes for the electrodes. Each cycle took 30 s with a loading rate of 1.2 MPa/s. All three samples tested were affected by the polarization effect, and it was minimized by removing the constant drift in the electrical signal to make the baseline of the FCR as horizontal as possible during signal processing. The corresponding results are presented in Fig. 2 (a), (b) and (c) respectively.

Figure 2 (c) only has nine cycles of data because the bond between the inner two electrodes and concrete failed during the test due to unexpected reasons. All samples except the third show a good correlation between FCR and loading with resistance generally increasing with increased compressive loading. This is the opposite of what might be expected as compression would tend to close up cracks orthogonal to the loading direction. It is hypothesised that it is the opening of cracks parallel to the loading due to Poisson expansion^9^ which is dominating the sensing performance, this will be further investigated in the subsequent numerical analysis. Closer investigation of Fig. 2 (b) shows a double frequency behaviour with resistance also increasing at minimum load presumably due to the opening of cracks orthogonal to the loading direction. In addition, the overall sensitivity of the first sample I appears to be lower than that of the second and third samples. Such variation is caused by the variation in the drilling process. To achieve the least destruction to the specimen’s structural integrity, the smallest possible hole should be drilled to embed electrodes. However, this was found to be tricky when the first sample was drilled, as smaller size driller heads heat up quickly due to the friction between themselves and the hard concrete. As a result, the ∅7 mm driller head was used for all specimens. This process induced more cracks to the first sample and therefore caused a variability in the sensing behaviour among the parallel samples. To reduce such variability, a better drilling technique is needed in the future, which includes using a small size driller head and an efficient cooling down method for the driller head during the drilling process.

### Numerical and mathematical model description and validation

#### Numerical investigation on the anisotropic conductivity

**Fig. 3:**
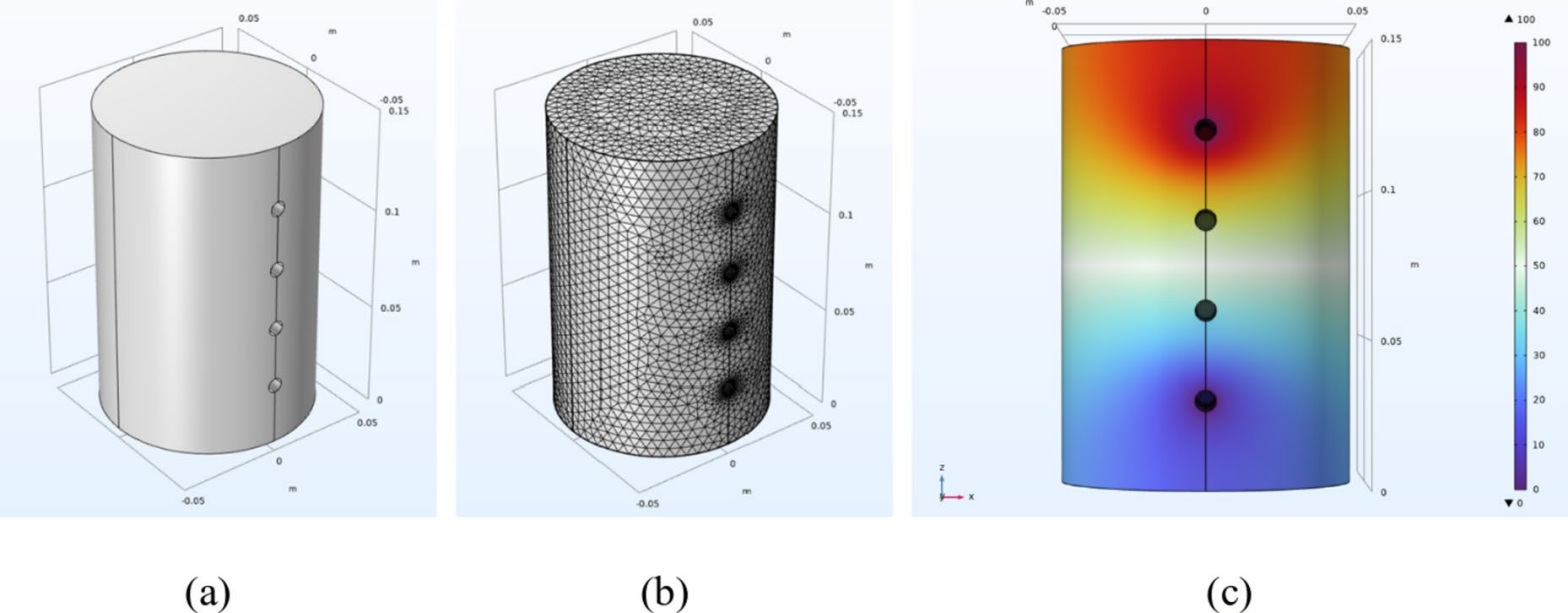
The numerical model of the concrete cylinder in COMSOL (**a**) the model, (**b**) the model with extra fine mesh for finite element analysis, (**c**) the voltage (V) distribution across the model.

To interpret the experimental results and understand the influence of the anisotropic conductivity due to crack opening on measured resistance, finite element analysis has been carried out using COMSOL Multiphysics^®^^10^. The numerical model created in COMSOL (Fig. 3 (a)) was identical to the geometries of the concrete cylinder samples tested. The concrete was assigned an electrical conductivity of 0.07 S/m in the direction of loading. In order to investigate the effects of Poisson expansion causing cracks to open normally to the applied compressive load, the electrical conductivity in the directions orthogonal to the loading was set to be smaller than that in the loading direction (axial for the cylinder). As the electrical conductivity of concrete highly depends on the number and size of the developed cracks, the horizontal conductivity $\:{C}_{h}$ and vertical conductivity $\:{C}_{v}$ were set at ratios of 0.01, 0.02, 0.05, 0.08, 0.1, 0.2, 0.3, 0.4, 0.5, 0.6, 0.7, 0.8, 0.9 and 1.

An extremely fine mesh was built for all the models and the AC/DC Module within COMSOL was utilised to calculate the current flow within the concrete specimen in response to a voltage applied across the outer two electrodes by solving Ohm’s Law. The current flowing between the outer electrodes was calculated by integrating the current density over the electrode surface and the potential difference between the inner pair of electrodes was then extracted from the analysis. There are three governing equations in the finite element calculation, i.e., Kirchhoff’s law of conservation of currents:

1 $$\:\nabla\:\cdot\:J=\:-\frac{\delta\:{\rho\:}_{s}}{\delta\:t}$$

where $\:J$ is the current density vector and $\:{\rho\:}_{s}$ is the space charge density.

Ohm’s law:

2 $$\:J=\:{\sigma\:}_{e}{E}_{e}$$

where $\:{\sigma\:}_{e}$ is the electric conductivity, and $\:{E}_{e}$ is the electrical field.

and electric potential $\:V$:

3 $$\:E=\:-\nabla\:\cdot\:V$$

An electrical potential of 100 V was supplied to the outer two electrodes of the sample, with voltage distribution results presented in Fig. 3 (c). Following the four-probe measurement method, the current flowing between the outer electrodes $\:I$, and the voltage between the inner two electrodes $\:V$ were extracted. The electrical resistance $\:R$ could be then calculated according to Eq. 4.

4 $$\:R=\:\frac{V}{I}$$

**Fig. 4:**
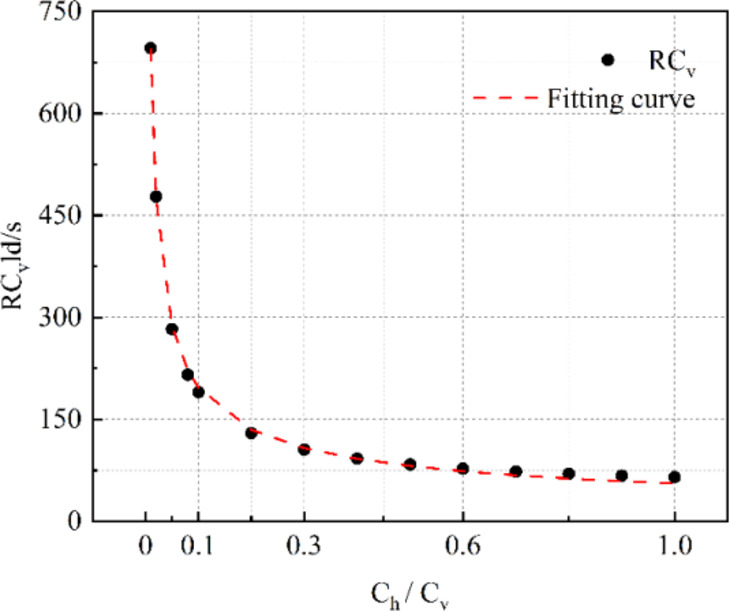
The relationship between the measured resistance $\:R\cdot\:{C}_{v}\cdot\:\frac{ld}{s}$ and $\:{C}_{h}/{C}_{v}$ from finite element analysis.

Figure 4 presents the variation of the resistance measured multiplied by the axial conductivity normalised by $\:ld/s$ to the $\:{C}_{h}$/$\:{C}_{v}$ ratio. The data was then fitted by a general power model using the Curve Fitting Tool in MATLAB.

Equations 5, 6 presents the curve fitting result with a R^2^ value of 0.999.

5 $$R \cdot C_{v} \cdot \frac{{ld}}{s} = 55.697~ \times ~\left( {\frac{{C_{h} }}{{C_{v} }}} \right)^{{ - 0.5478}}$$

where $\:l$ is the electrode penetration, d is the electrode diameter.

Hence:

6 $$\:R=4.774\:{{C}_{v}}^{-0.4522}{{C}_{h}}^{-0.5478}$$

This indicates that for the geometry of the sample tested, the measured resistance $\:R$ is almost equally affected by changes in resistance in the axial and orthogonal directions. Compression in the axial direction will lead to orthogonal cracks initially closing giving a modest decrease in resistance but subsequent opening of parallel cracks due to Poisson expansion will then lead to a substantial increase in overall resistance as the large decreases in horizontal conductivity due to crack opening will dominate the small changes in conductivity in the direction parallel to the load due to axial compression of intact material. At modest loads, parallel cracks in the specimen will constrain current to flow in a small volume of material between the electrodes leading to an increased resistance relative to a homogenous material in which current will flow in a larger volume.

#### Mathematical model description and validation

In order to further investigate the effects of Poisson expansion on measured behaviour, the resistivity of the samples in different directions can be linked to the variation of resistivity with strain in tension and compression.

The Gauge Factor (GF) for an electrical strain gauge in an isotropic material can be defined as^11^:

7 $$\:GF=\frac{\frac{\varDelta\:R}{R}}{}=\:\frac{\frac{\varDelta\:\rho\:}{{\rho\:}_{0}}}{}+(1+2\nu\:)$$

where $\:\varDelta\:\rho\:$ is the small change in electrical resistivity, $\:{\rho\:}_{0}$ is the original resistivity,* ε* is the strain and $\:\nu\:$ is the Poisson’s ratio. According to different existing studies, the reported values of the gauge factor of concrete vary in a wide range from 0.35^12^ to over 6000^13^, depending on if it is with or without electrically conductive fillers. For gauge factor for plain concrete, the values have been reported as low as nearly 0 to as high as over 200. For example, Zhang et al.^14^ reported a gauge factor of 70 for plain mortar made of OPC 42.5R with a w/c ratio of 0.42; Birgin et al.^15^ reported a gauge factor of 60 for CEMI 42.5R paste with a 0.5 w/c ratio and Ding et al.^16^ found a gauge factor of 21.5 for CEMI 52.5 cement paste with a w/c ratio of 0.4. These values indicating strain sensitivities can be increased by adding electrically conductive fillers. For instance, Han et al.^17^ reported the gauge factor was increased from 0.3 to 704 by adding electrostatic self-assembled carbon nanotube and nano carbon black composite fillers into pure cement mortar. Similarly, Belli et al.^18^ found a gauge factor of 613.5 for carbon fibre embedded cement-based mortars. The gauge factor of this concrete samples in this study is around 200, which will be discussed later in this section. Poisson’s ratio is the ratio of transverse contraction strain to longitudinal extension strain in the direction of stretching force^19^. It affects the resistance owing to the change in cross sectional area of the sample and is theoretically often applied in the elastic range of concrete. Consideration of post-cracking Poisson’s ratio of concrete can be significant in certain cases^20^ as damage and micro-cracking can affect the Poisson’s ratio. This study takes a phenomenological approach and thus refers to an ‘equivalent Poisson’s ratio’ for mathematical investigation.

Expressing Eq. 7 in terms of conductivity leads to:

8 $$\:{C}_{v}=\:{C}_{0}\cdot\:\:\frac{1}{1+\:{\epsilon\:}_{v\:}[{GF}_{v}-\left(1+2\nu\:\right)]}$$

9 $$\:{C}_{h}=\:{C}_{0}\cdot\:\:\frac{1}{1+\:{\epsilon\:}_{h\:}[{GF}_{h}-\left(1+2\nu\:\right)]}$$

where the subscripts $\:v$ and $\:h$ represent the property in the vertical and horizontal direction respectively.

As:

10 $$\:{\nu\:}_{c}=\:-\frac{{\epsilon\:}_{h}}{{\epsilon\:}_{v}}$$

This then gives the horizontal conductivity $\:{C}_{h}$:

11 $$\:{C}_{h}=\:{C}_{0}\:\cdot\:\:\frac{1}{1-\:{\epsilon\:}_{v}\nu\:[{GF}_{h}-\left(1+2\nu\:\right)]}$$

The finite element analysis results shown in Eq. 6 shows that

12 $$\:R=\:\alpha\:\cdot\:{{C}_{h}}^{n}{{C}_{v}}^{-(1+n)}$$

Therefore, the derivative of $\:R$ with respect to vertical strain is:

13 $$\frac{{dR}}{{d\varepsilon _{v} }} = ~\alpha \cdot \left[ {n \cdot C_{h} ^{{n - 1}} C_{v} ^{{ - \left( {1 + n} \right)}} \cdot \left( {\frac{{dC_{h} }}{{dC_{v} }}} \right) - \left( {1 + n} \right) \cdot C_{h} ^{n} C_{v} ^{{ - \left( {2 + n} \right)}} \cdot \left( {\frac{{dC_{v} }}{{d\varepsilon _{v} }}} \right)} \right]$$

Taking derivatives of $\:{C}_{v}$ and $\:{C}_{h}$ from Eqs. 8 and 10:

14 $$\:\frac{d{C}_{v}}{d{\epsilon\:}_{v}}=\:-\frac{{C}_{0}\cdot\:[{GF}_{v}-\left(1+2\nu\:\right)]}{{\{1+\:{\epsilon\:}_{v}\cdot\:\left[{GF}_{v}-\left(1+2\nu\:\right)\right]\}}^{2}}$$

15 $$\:\frac{d{C}_{h}}{d{\epsilon\:}_{v}}=\:-\frac{\nu\:{C}_{0}\cdot\:[{GF}_{h}-\left(1+2\nu\:\right)]}{{\{1-\:{\nu\:\epsilon\:}_{v}\cdot\:\left[{GF}_{v}-\left(1+2\nu\:\right)\right]\}}^{2}}$$

Since the Poisson’s ratio $\:\nu\:$ is much smaller than the $\:GF$ (~ 200) in our case, the term of $\:\left(1+2\nu\:\right)$ in Eqs. 14 and 15 becomes insignificant and thus is ignored in the following calculations. Combining Eqs. 14, 15 and 13,

16 $$\frac{{dR}}{{d\varepsilon _{v} }} = ~\alpha \cdot \left[ {n \cdot C_{0} \cdot C_{h} ^{{n - 1}} \cdot C_{v} ^{{ - \left( {1 + n} \right)}} \cdot \frac{{\nu \cdot GF_{h} }}{{\left( {1 - \nu GF_{h} } \right)^{2} }} + \left( {1 + n} \right) \cdot C_{0} \cdot C_{h} ^{n} \cdot C_{v} ^{{ - \left( {2 + n} \right)}} \cdot \frac{{GF_{v} }}{{\left( {1 + ~\varepsilon _{v} GF_{v} } \right)^{2} }}} \right]$$

To set a boundary condition beyond which the measured electrical resistance $\:R\:$begins to be dominated by the material behaviour in the horizontal direction, the $\:\frac{dR}{d{\epsilon\:}_{v}}$ in Eq. 16 needs to be 0. Thus:

17 $$\:\alpha\:\cdot\:\left[n\cdot\:{C}_{0}\cdot\:{{C}_{h}}^{n-1}\cdot\:{{C}_{v}}^{-\left(1+n\right)}\cdot\:\frac{\nu\:\cdot\:{GF}_{h}}{{\left(1-v{\epsilon\:}_{v}{GF}_{h}\right)}^{2}}+\left(1+n\right)\cdot\:{C}_{0}\cdot\:{{C}_{h}}^{n}\cdot\:{{C}_{v}}^{-\left(2+n\right)}\cdot\:\frac{{GF}_{v}}{{\left(1+\:{\epsilon\:}_{v}{GF}_{v}\right)}^{2}}\right]=0$$

Rearrangement of Eq. 17 gives:

18 $$\:n\cdot\:\nu\:\cdot\:\left(\frac{{C}_{v}}{{C}_{h}}\right)\cdot\:\frac{{GF}_{h}}{{\left(1-\nu\:{\epsilon\:}_{v}{GF}_{h}\right)}^{2}}=\:-(1+n)\cdot\:\frac{{GF}_{v}}{{\left(1+\:{\epsilon\:}_{v}{GF}_{v}\right)}^{2}}$$

As $\:{\epsilon\:}_{v}$ is small, the terms of $\:\nu\:{\epsilon\:}_{v}{GF}_{h}$ and $\:{\epsilon\:}_{v}{GF}_{v}$ in Eq. 18 can be ignored. Since the material is initially isotropic, for small $\:{\epsilon\:}_{v}$, $\:\frac{{C}_{v}}{{C}_{h}}$ can be taken as 1. This gives the threshold ratio of $\:{GF}_{v}$ to $\:{GF}_{h}$ being:

19 $$\:\frac{{GF}_{v}}{{GF}_{h}}=\:-\left(\frac{n}{1+n}\right)\cdot\:\nu\:$$

The vertical and horizontal gauge factors can be better thought of as being those in compression and tension respectively and hence $\:{GF}_{h}$ would be expected to be substantially larger than $\:{GF}_{v}$ due to cracks opening in tension. Equation 19 implies that under axial compression measured axial resistance will increase due to vertical crack opening, if:

20 $$\:{GF}_{tensile}>-\left(\frac{1+n}{n}\right)\cdot\:\frac{1}{\nu\:}\cdot\:{GF}_{vcompressive}$$

The Poisson’s ratio of concrete is usually taken around 0.2^21^ and the coefficient $\:n$ from numerical modelling results presented in Eq. 5 is $\:-0.5478$. Therefore, according to Eq. 20, if $\:{GF}_{tensile}$ is larger than 4.13$\:{GF}_{compressive}$, the measured resistance will present a positive correlation between the FCR and the compressive load.

**Fig. 5:**
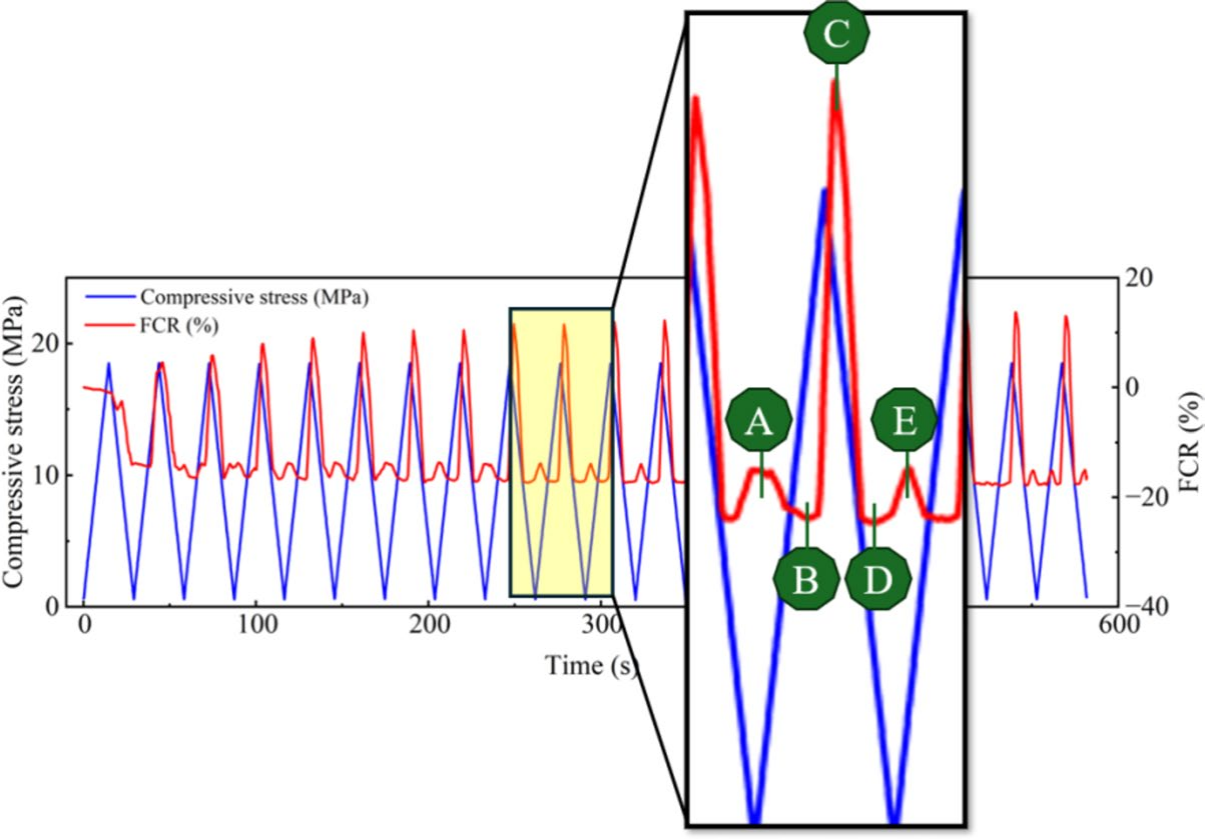
Magnified loading cycles from Fig. 2(b).

This mathematical model can thus be used to demonstrate the experimental result of the concrete cylinder test shown in Fig. 2 (b) which is magnified and presented in Fig. 5. The magnified area shows a typical loading cycle of the sample, where point A marks the start of the cycle; point C marks the time when maximum compression was reached; and point E marks the end of the cycle. At very small strains, i.e., from point A to B in Fig. 5, the sample displayed isotropic behaviour, i.e., $\:{GF}_{h}$ = $\:{GF}_{v}$ giving a negative correlation between compression and FCR. When the compressive load increased beyond point B, longitudinal cracks opened due to Poisson expansion, increasing the orthogonal (tensile) gauge factor above the threshold defined by Eq. 19, and thus the sample’s resistance increased with increasing axial loading from point B to D. After point D, the stains became small again and the correlation between compression and FCR recovered back to negative till the end of the loading cycle (point E).

This finding is significant as it could be used to assess crack development and differentiate the elastic and plastic stages of the sample for structural health monitoring purposes. To be more specific, if the correlation between the compressive load and FCR reverses from negative to positive, it could indicate the presence of substantial cracking within the concrete structure and thus maintenance and repair might be needed.

#### Numerical investigation on effects of sample geometry and electrode placement

To investigate the effect of the electrode penetration (l) – spacing (s) ratio l/s on the power coefficient $\:n$ in the $\:R\cdot\:{C}_{v}$ - $\:{C}_{h}/{C}_{v}$ relationship described in Eq. 12, three different specimens were modelled in COMSOL, i.e., a cube (50 mm $\:\times\:$ 50 mm $\:\times\:$ 50 mm), a prism (100 mm $\:\times\:$ 100 mm $\:\times\:$ 150 mm) and a cylinder (diameter 100 mm, height 150 mm).

**Fig. 6:**
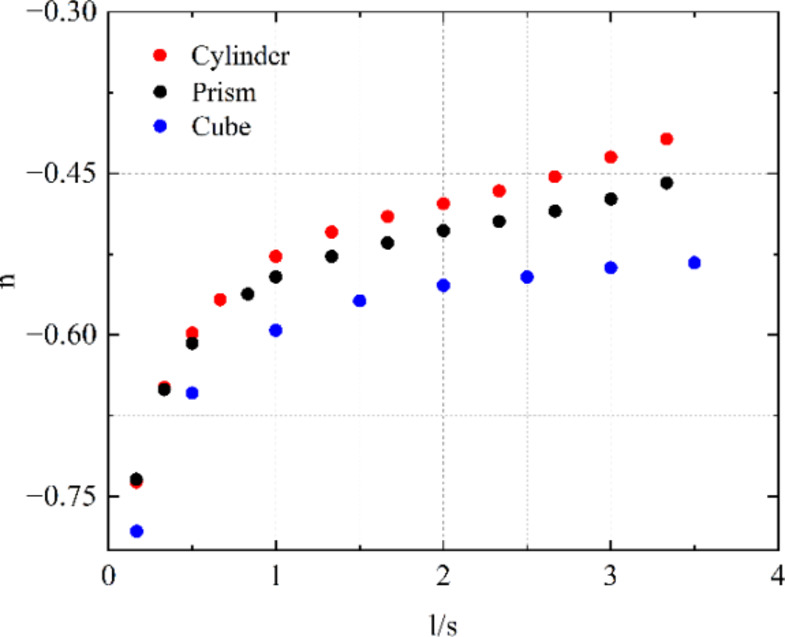
Finite element results of the relationship between the electrodes’ penetration to spacing ratio l/s and power coefficient $\:n$ of cube, prism, and cylinder.

Figure 6 presents the finite element modelling results of the relationship between the electrodes’ penetration to spacing ratio l/s (horizontal axis) and power coefficient $\:n$ (vertical axis) of the cube, prism, and cylinder models. For all three types of models, the power coefficient $\:n$ increases with increasing electrode penetration to spacing ratio l/s, but there is only a modest effect of sample geometry, as might be expected if the electrode spacing is small relative to the sample size. As the l/s ratio increases the value of n increases and the term $\:-(1+n)/n$ in Eq. 19 will increase. The effect of Poisson crack opening on resistance will thus be more pronounced for electrodes which are short relative to their spacing. Surface-mounted electrodes with limited penetration will thus be most effective in measuring the presence of cracks within the concrete, conforming with the results of the authors’ previous experimental and FEA study on cementitious composites under flexure, where it was found that to obtain accurate measurements, the electrodes need to be at close spacings with shallow penetration^22^.

## Conclusion

This paper, for the first time, looks beyond the commonly discussed resistance-based and capacitance-based self-sensing mechanisms only in the elastic deformation regime of the concrete and explores the sensing behaviour of as-built plain concrete with micro-cracks in the material. The measurement technique to achieve intrinsic self-sensing on as-built regular concrete (i.e. without conductive fillers) by drilling the concrete to insert conductive epoxy for electrode embedment was tested. The Poisson effect of the cracks’ expansion on the correlation of the fractional change in electrical resistivity and the compressive load of concrete was investigated. The work underpins the possibility of achieving self-sensing on regular, existing concrete structures, without the need of embedding electrically conductive fillers.

Experimental results on 14-month-old plain concrete showed effective sensing behaviours, driven by the opening of cracks parallel to the loading direction due to Poisson expansion. Numerical simulation of the concrete sample further validated this theory by creating a finite element model with different electrical conductivity in parallel and orthogonal directions by proxy. It was found that the correlation between the FCR and the compressive load depends on the relationship between the tensile and compressive gauge factors. Once cracks begin to open due to Poisson expansion, the resistance reflects the material’s behaviour in the direction orthogonal to the loading direction. Extended finite element analysis further found that the threshold condition is strictly related to the sample geometry and its electrode layout. To the best of the authors’ knowledge, self-sensing through the Poisson expansion effect on crack openings on existing plain concrete has not yet been explored in the literature. While concrete without conductive additives is thought of as being an insulator for capacitance-based self-sensing, measuring changes in its electrical properties is achievable also through the Poisson expansion effect, and it only requires instrumentation sensitive enough to measure very small currents with modest voltages. The fractional changes in resistivity exhibited by the concrete are sufficient for self-sensing even without conductive additives. Further investigation needs to be carried out to study how to best apply the technique proposed in this paper practically to real-life concrete structures.

## Data Availability

The datasets used and/or analysed during the current study available from the corresponding author on reasonable request.
